# Experimental and constitutive model study on the mechanical properties of a structural plane of a rock mass under dynamic disturbance

**DOI:** 10.1038/s41598-022-25544-0

**Published:** 2022-12-08

**Authors:** Yongjiang Yu, Yuntao Yang, Jingjing Liu, Pengbo Wang, Shipeng Zhang, Zhenmeng Wang, Shangqing Zhao

**Affiliations:** College of Mining Engineering, Liaoning Technical University, Fuxin, 123000 Liaoning China

**Keywords:** Engineering, Civil engineering

## Abstract

An accurate description of the mechanical properties and deformation characteristics of a structural plane of a rock mass with a large chamber or slope under the ultimate stress with periodic stress disturbances is of great significance to ensure the stability and safety of underground rock engineering. By theoretically analysing the strength effect of a structural plane of a rock mass under dynamic disturbance, a criterion for the occurrence of shear damage on a structural plane of a compressed rock mass under dynamic disturbance is proposed. The results of the cyclic disturbance kinetic test show that there is a disturbance threshold for the shear failure of the structural plane under different disturbance stresses. When the disturbance stress is lower than the disturbance threshold, the cumulative plastic strain stabilizes with an increasing number of cycles; when the disturbance stress is higher than the disturbance threshold, an S-shaped curve of cumulative plastic strain versus the number of cycles is observed, revealing the progressive damage process and mechanism of such a rock structure plane under periodic dynamic disturbance. Based on perturbation concept theory, the relationship between the accumulated plastic strain and the number of cyclic loadings is similar to the relationship between strain and time, the creep curve. A new nonlinear viscous element is proposed, and the nonlinear element and the deformation element considering structural plane closure and sliding are combined with the Burgers model to form an 8-element nonlinear viscoelastic‒plastic creep constitutive model. Using the global optimization algorithm of 1stOpt, model validation and parameter identification are performed on the experimental data, and the results show that the model curve has a very good agreement with the experimental data. The model can accurately reflect the deformation characteristics of a structural plane of a rock mass under periodic dynamic disturbance. These research results provide a new idea for analysing disturbance-induced geohazards.

## Introduction

Structural planes such as joints, fissures, and weak interlayers exist in natural rock masses. The mechanical properties of the structural planes of a rock mass are often the factors controlling the safety of rock mass engineering^1–3^. The total deformation of a structural plane of a rock mass includes not only the deformation of the intact rock mass but also the closure deformation and shear deformation of the structural plane under compression and exhibits different mechanical properties such as elasticity, plasticity, and viscosity, under an external load. In particular, when the structural plane of a deep, large chamber or slope rock mass is subjected to periodic dynamic disturbance under a high-stress state close to the ultimate strength of the rock, the mechanical characteristics and failure modes are more complicated. Therefore, the deformation and failure mechanism of the deep structural plane of a rock mass under a cyclic dynamic disturbance and the accurate construction of a constitutive model are key scientific problems for the safety of rock mass engineering under dynamic disturbance.

To study the damage mechanism of the structural plane of a rock mass under the action of cyclic load disturbance, Liu et al.^4^ investigated the mechanism of dynamic collapse instability of large structural planes under the action of stress waves, established a mechanical model for shear slip instability of structural planes, derived an energy criterion and a stress criterion for the shear slip of structural planes, and derived conditions for the shear instability of structural planes. Sun et al.^5^ conducted a perturbed rheological test of a muddy soft interlayer under the intermittent cyclic action of dynamic shear, and the results of the study showed that a stress state threshold value exists when the specimen finally undergoes rheological damage under the intermittent perturbation of cyclic dynamic shear, which is determined by the sum of the static shear stress and peak dynamic shear stress. Liu et al.^6^ studied the cumulative damage characteristics of the structural planes of rock masses under different damage modes and analysed the influence law of the number of cyclic loadings, loading rates, and amplitudes on the shear characteristics of the structural plane of rock masses. Deng et al.^7^ used granular flow software to simulate the deformation characteristics of a structural plane rock mass under cyclic loading in before the peak stress and compared it with indoor experiments, and concluded that the magnitude of the cyclic loading is the key factor to determine the cumulative damage rate of a structural plane rock mass Zhang et al.^8^ studied the normal cyclic loading deformation characteristics of natural structural planes with a certain thickness and concluded that when the normal stress was within 20 MPa, the normal stress-closing deformation relationship of the structural plane could be better simulated by using a hyperbolic model and a modified hyperbolic model considering the correction parameters at different loading stages. Lu et al.^9^ showed that axial strain, circumferential strain, and volume deformation all play an important role in the deterioration damage of yellow sandstone and that the cycle number-maximum stress curve could be used to predict the fatigue life of the rock to some extent. Zhu et al.^10^ concluded that cyclic loading conditions have significant effects on the plastic properties and fatigue damage characteristics of rock masses, and revealed the fatigue damage evolution process and macroscopic rupture characteristics by performing acoustic emission tests. To study the constitutive model of a structural plane, Wang et al.^11^ used damage mechanics theory to establish the damage evolution model and damage intrinsic model of jointed rock masses by considering the structural effect of rock masses and the coupling effect of loads. Zhao et al.^12^ formed a six-element nonlinear viscoelastic‒plastic creep model by connecting a nonlinear element and a Burgers creep model in series to realize the simulation of the accelerated creep stages of rock masses containing weak structural planes. Li et al.^13^ introduced a nonlinear viscous accelerating element and material damage variables applicable to structural planes to establish a shear creep damage intrinsic model for a nonpenetrating structural plane. Deng et al.^14^ established the ontological model of tensile and shear damage of structural planes of rock masses and a damage criterion of tensile and shear damage based on the energy principle. Yang et al.^15^ used a damage model considering the effects of the nodal normal vector and area density to describe the discontinuity of the joints and verified the validity of the model with FLAC^3D^. Zhou et al.^16^ proposed a statistical damage model using the Weibull distribution, which takes into account joint orientation by incorporating the Jaeger and modified Hoek‒Brown failure criteria for jointed rock masses. Yang et al.^17^ conducted graded loading and unloading uniaxial creep tests on sandstone and established a creep damage model. Based on the cyclic triaxial loading and unloading creep phenomenon of rock, He et al.^18^ conducted shear creep tests on two types of marble containing soft and hard structural planes and then proposed a time-varying statistical damage intrinsic model to describe the time-varying damage properties based on fractional order calculus theory and damage evolution law. Peng et al.^19^ proposed a joint plastic fatigue component and a double-triggered nonlinear viscous fatigue composite component and then established a new elastic‒plastic viscous fatigue model of jointed rock.

In summary, there are relatively few studies on the degradation of the shear strength of the structural plane and constitutive models under periodic dynamic disturbances. Most of the existing studies are based on damage statistical theory. The damage constitutive models constructed cannot fully characterize the different deformation stages of the structural plane under dynamic disturbance and do not consider the impact of the disturbance threshold on the deformation failure process of the structural plane under the ultimate strength. We intend to determine the number of loading and unloading cycles and the variation in the cumulative plastic strain through disturbance dynamics experiments under cyclic loading and construct a cyclic loading‒unloading constitutive model for a rock mass under the influence of the disturbance threshold. The research results provide a theoretical basis for the effective prevention and control of geological disasters caused by engineering activities and new ideas for analysing the mechanism of geological disasters induced by disturbances.

## Strength effect of the structural plane under disturbance action

**Figure 1:**
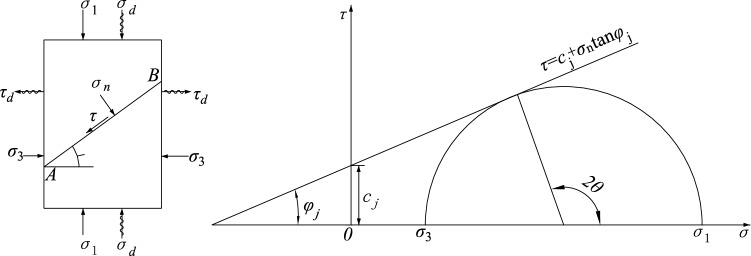
Stress state of a structural plane in rock under dynamic disturbance.

A deep unexcavated rock mass is in a triaxial stress state under the combined effects of the self-weight stress and tectonic stress field, and large chambers or slopes formed by excavation lead to a redistribution of stresses, leaving the geological structure in a state of biaxial compression due to one side facing a void. When subjected to periodic dynamic disturbances the stress state of the structural plane is the result of the combined effect of the original and disturbance stress states. For this reason, it is assumed that there is a blasting stress wave acting on the engineering rock so that the structural plane is subjected to radial compressional and tangential tensile waves. Figure 1 shows the stress state of the structural plane under dynamic disturbance.

According to the theory of the Mohr stress circle, the positive and shear stresses acting on the AB surface of the structure are

1 $$\left\{ \begin{gathered} \sigma_{n} = \frac{{1}}{{2}}\left( {\sigma_{{1}} + \sigma_{{3}} + \sigma_{d} - \tau_{d} } \right) + \frac{{1}}{{2}}\left( {\sigma_{{1}} + \sigma_{d} - \sigma_{{3}} + \tau_{d} } \right)\cos \left( {2\theta } \right) \hfill \\ \tau = \frac{{1}}{{2}}\left( {\sigma_{{1}} + \sigma_{d} - \sigma_{{3}} + \tau_{d} } \right)\sin \left( {2\theta } \right) \hfill \\ \end{gathered} \right.$$

${\sigma }_{1}$ is the longitudinal stress, ${\sigma }_{3}$ is the lateral stress, ${\sigma }_{d}$ are the radial compressional waves, ${\tau }_{d}$ are the tangential tensile waves, and $\theta$ is the discontinuity inclination.

The structural plane shear stress intensity curve obeys the Coulomb criterion

2 $$\tau { = }c_{j} + \sigma_{n} \tan \varphi_{j}$$

*C*_*j*_ is the cohesive force of the structural plane, and ${\varphi }_{j}$ is the internal friction angle of the structure surface.

Based on Eqs. (1) and (2), the criterion for generating slip damage along structural plane AB is obtained

3 $$\sigma_{{1}} = \frac{{2\left[ {c_{j} + \left( {\sigma_{3} - \tau_{d} } \right)\tan \varphi_{j} } \right]}}{{\left( {1 - \tan \varphi_{j} \cot \theta } \right)\sin (2\theta )}}{ + }\sigma_{d} - \sigma_{3} + \tau_{d}$$

**Figure 2:**
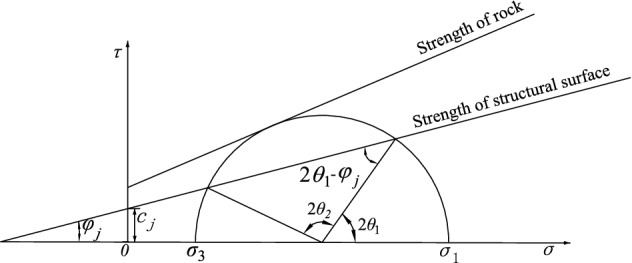
Range of discontinuity dip.

It can be seen from the above equation that ${\sigma }_{3}$, ${\sigma }_{d}$, ${\tau }_{d}$, ${c}_{j}$ and $tan{\varphi }_{j}$ must be known. ${\sigma }_{1}$ is symmetric about $\theta =\pi /2$. When $\theta =\pi /2$, $\theta \to {\varphi }_{j}$, and $0<\theta <{tan}^{-1}\varphi$, the structure plane undergoes self-locking, ${\sigma }_{1}\to \infty$, and the principal stress required for sliding to occur on the structural plane is infinite. That is, this combination is unlikely to result in structural plane damage, but along another other direction, shear damage may form through the combination of materials. Figure 2 shows the range of dip angles along the structural plane of the damage.

From the geometric relationship, we know that

4 $$\theta_{{1}} = \frac{{\varphi_{j} }}{2} + \frac{1}{2}\arcsin \left[ {\frac{{\left( {\sigma_{1} + \sigma_{d} + \sigma_{3} - \tau_{d} + 2c_{j} \cot \varphi_{j} } \right)\sin \varphi_{j} }}{{\sigma_{1} + \sigma_{d} - \sigma_{3} + \tau_{d} }}} \right]$$

5 $$\theta_{2} = \frac{\pi }{2} + \frac{{\varphi_{j} }}{2} - \frac{1}{2}\arcsin \left[ {\frac{{\left( {\sigma_{1} + \sigma_{d} + \sigma_{3} - \tau_{d} + 2c_{j} \cot \varphi_{j} } \right)\sin \varphi_{j} }}{{\sigma_{1} + \sigma_{d} - \sigma_{3} + \tau_{d} }}} \right]$$

Derive Eq. (3) in terms of $\theta$, letting the first-order derivative be zero, that is, the condition that satisfies the minimum value ${\sigma }_{1,min}$ obtained by ${\sigma }_{1}$ is

6 $$\tan 2\theta = - \cot \varphi_{j}$$

Based on Eqs. (3) and (6),

7 $$\sigma_{{\text{1,min}}} = \frac{{2\left[ {c_{j} + \left( {\sigma_{3} - \tau_{d} } \right)\tan \varphi_{j} } \right]}}{{\sqrt {1 + \tan^{2} \varphi_{j} } - \tan \varphi_{j} }} + \sigma_{d} - \sigma_{3} + \tau_{d}$$

From the above analysis, it can be seen that the structural plane strength under dynamic and static loading is mainly determined by ${\sigma }_{3}$, ${\sigma }_{e}$, ${\sigma }_{d}$, ${\tau }_{d}$, ${c}_{j}$ and $\mathrm{tan}{\varphi }_{j}$. The damage mode of the specimen is determined by the stress state, the structural plane strength, and the dip angle of the structural plane. Whether structural control damage occurs depends primarily on the effect of ${\sigma }_{3}$, ${\sigma }_{e}$, ${\sigma }_{d}$ and ${\tau }_{d}$ on $\theta$. Structural control damage may occur only when ${\mathrm{tan}}^{-1}\varphi <\theta <\pi /2$ and ${\theta }_{1}<\theta <{\theta }_{2}$.

## Test system and plan

### Sample preparation

**Figure 3:**
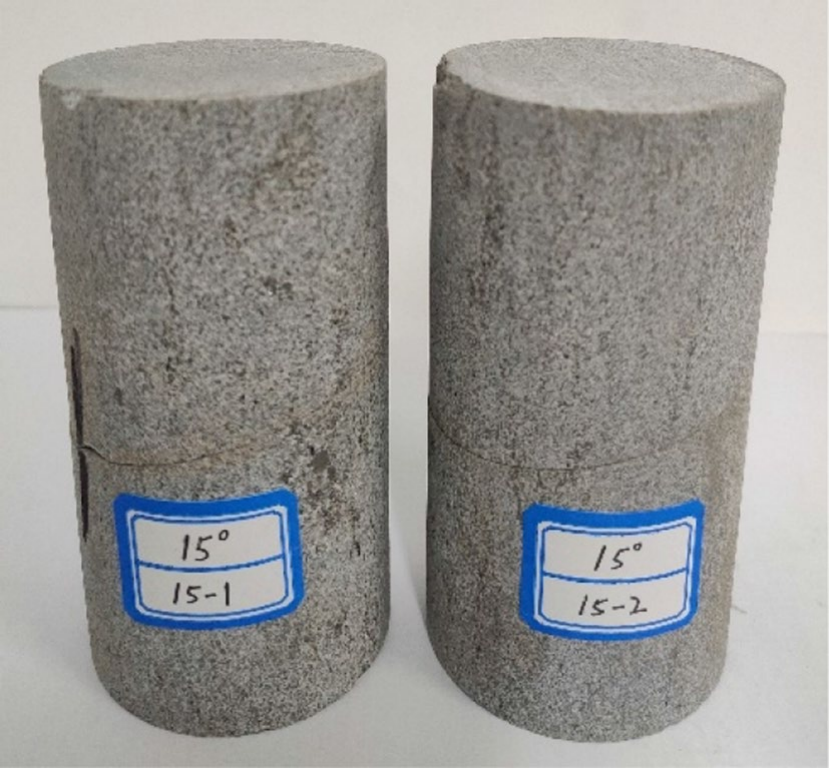
Rock test pieces.

To reduce the influence of rock discreteness on the test results, one large-scale homogeneous coarse-grained sandstone rock block from the Ping Shuo second coal well of China Coal was selected as the test material. First, a core sample was taken by a sampling machine from the rock sample and processed into a cylindrical test piece with a diameter of 50 mm and a height of 100 mm. The two ends and sides of the test piece were carefully ground, and the nonparallelism and nonperpendicularity were lower than 0.02 mm; the surface was smooth with no obvious defects. Finally, the cylindrical specimens of coarse-grained sandstone were cut by a multiangle cutting machine. According to the geological conditions of the mine, the dip angle of the coal seam is approximately 15°. Thus, in this experiment, the inclination angle chosen for the structural plane was 15°, so the cutting angle was adjusted to 15°. After cutting, rock specimens for which the sum of the angles of the coarse-grained sandstone was 90° were combined. The structural plane was bonded with epoxy resin adhesive, which allows the structural plane to have a certain strength without greatly affecting the physical and mechanical properties of the jointed rock mass (see Fig. 3).

### Testing instruments and plans

**Figure 4:**
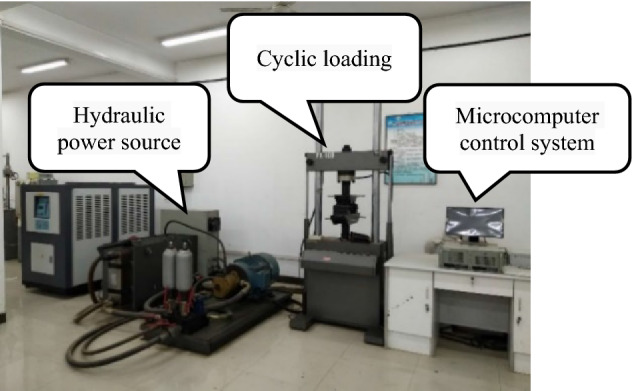
Testing instrument.

**Figure 5:**
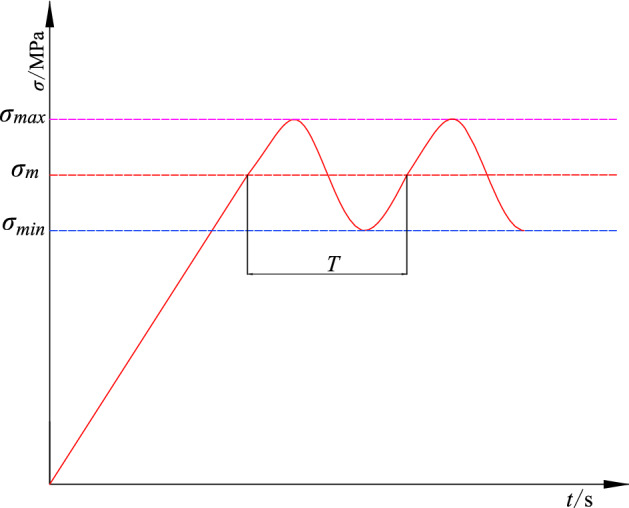
Schematic of the wave for the loading cycle.

The tests were carried out on a PA-100 microcontrol electrohydraulic servo fatigue testing machine at the Mechanical Testing Center of Liaoning Technical University. The test system is shown in Fig. 4. The maximum static load of the tester is 50 kN, the maximum dynamic test force is ± 50 kN, and the disturbance frequency can range from 0.01 to 30 Hz. To perform cyclic dynamic disturbance testing of a rock under a certain static force, first, the static load is applied to the rock by a static loading method at a certain loading speed. After the stress reaches a predetermined value, the static stress at this point is used as the average stress, and a cyclic dynamic load is applied. Load control is used to maintain a constant loading rate during cyclic disturbances. To simulate the elastic wave from vibration propagation, the cyclic disturbance wave is a sinusoidal wave, and the test machine cyclically applies the load on the rock with a constant upper limit load and lower limit load until the rock breaks. The loading process and characteristics are shown in Fig. 5, where $\sigma_{\max }$ is the upper limit stress of the cyclic load, $\sigma_{\min }$ is the lower limit stress of the cyclic load, $\Delta \sigma = \sigma_{\max } - \sigma_{m}$ is the dynamic disturbance amplitude, and *T* is the loading cycle. During the test, the test system can collect the axial load, axial deformation, lateral deformation, and time data and draw corresponding parameter relationship curves.

**Figure 6:**
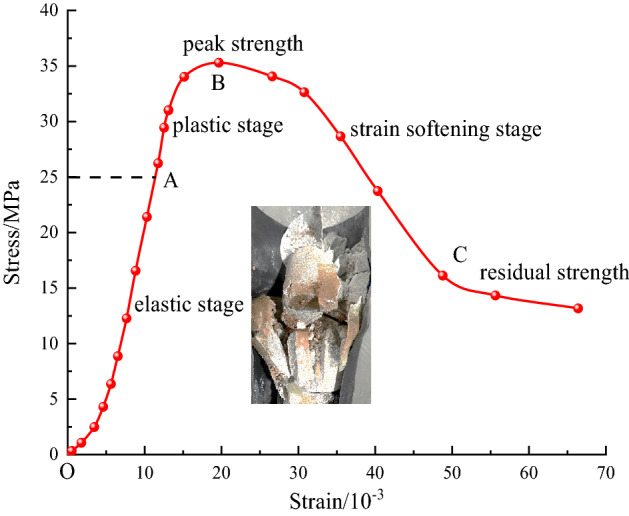
Stress‒strain curves under uniaxial compression.

To obtain reasonable values for ${\sigma }_{m}$, first, uniaxial compression testing of the 15° structural plane of the rock was carried out to obtain the full curve for the uniaxial static stress‒strain of the structural plane of the rock, as shown in Fig. 6. OA is the elastic stage, AB is the plastic stage and BC is the strain softening stage. Under the action of a small cyclic perturbation, if ${\sigma }_{m}$ takes the value of the OA section, plastic damage will not occur on the structural plane of the rock. If ${\sigma }_{m}$ takes the value of section AB, the structural plane of the rock is destroyed too fast, and it is difficult to collect valid experimental data. In summary, the stress corresponding to point A was chosen as the average value of the cyclic loading stress, $\sigma_{m} = 25\;{\text{MPa}}$. The perturbation amplitudes were selected to be $5\;{\text{MPa}}$, $11\;{\text{MPa}},$ and $15\;{\text{MPa}}$. When $\Delta \sigma = 5\;{\text{MPa}}$, the value of $\sigma_{\max }$ is in the range of plastic deformation; when $\Delta \sigma = 11\;{\text{MPa}}$, the value of $\sigma_{\max }$ is equal to the peak strength; when $\Delta \sigma = 15\;{\text{MPa}}$, the value of $\sigma_{\max }$ is greater than the peak strength. The three perturbation amplitudes have special characteristics. Then, the axial cyclic disturbance load of the sine wave waveform with a fixed frequency of 2 Hz was applied to the test piece. The specific test plan is shown in Table 1.

**Table 1:** Test plan.

No.	Number of test pieces	Stress/MPa	Frequency/Hz	Amplitude /MPa	*σ*_max_/MPa	*σ*_min_/MPa	*σ*_*m*_/MPa
N1	5	25	2	5	30	20	25
N2	5	25	2	11	36	14	25
N3	5	25	2	15	40	10	25

## Analysis of test results

### Variation in the stress‒strain curve during the dynamic disturbance

**Figure 7:**
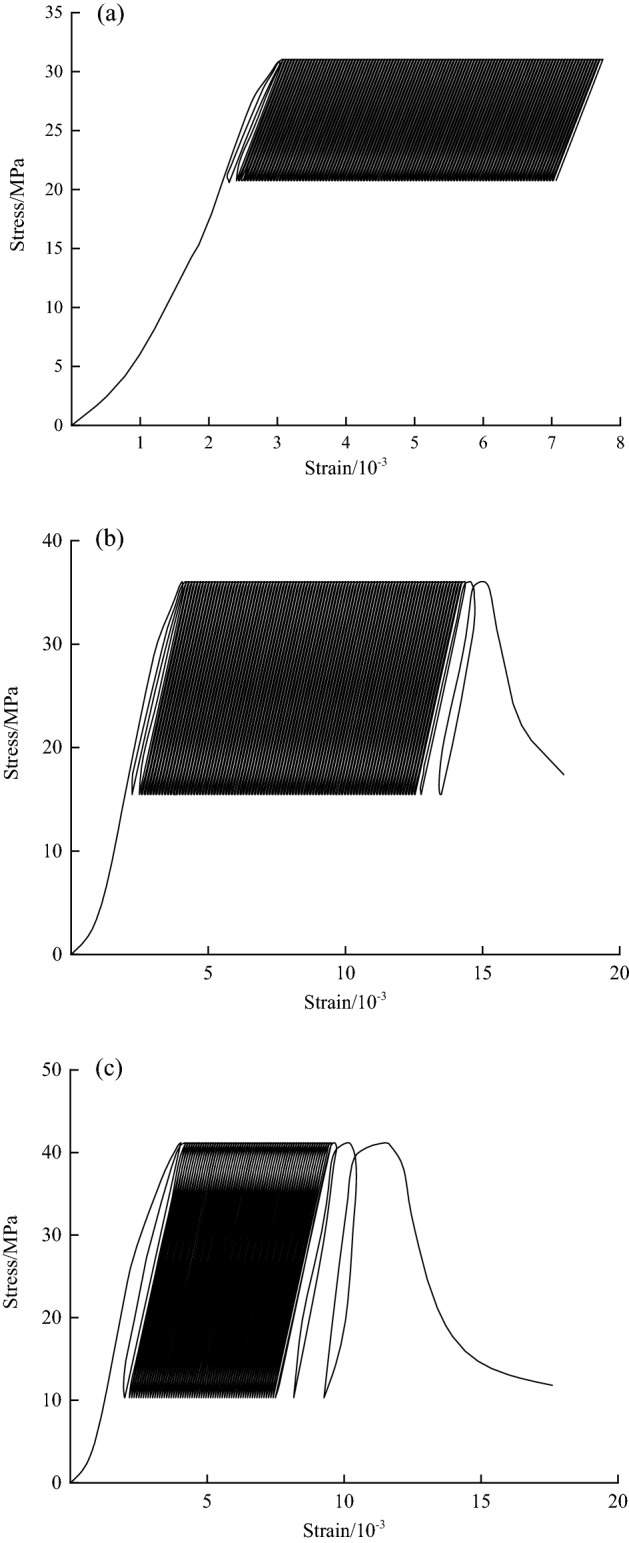
Cyclic loading stress‒strain curves at different stress amplitudes: (**a**) $\Delta \sigma =5MPa$, (**b**) $\Delta \sigma =11MPa$, (**c**) $\Delta \sigma =15MPa$.

The test data processing used to obtain the stress‒strain curves under cyclic loading at different stress amplitudes is shown in Fig. 7. As seen from Fig. 7, the strain exhibits a hysteresis effect relative to the stress, and a hysteresis curve appears with each loading and unloading process, which is characterized by three stages of sparse-dense-sparse as the number of cycles increases, and the hysteresis loop gradually approaches the direction of increasing strain. When the disturbance stress amplitude is $\Delta \sigma = 5\;{\text{MPa}}$, after 120 cycles of loading, the accumulated plastic strain of the specimen hardly changes, and the rock specimen is not damaged. When the disturbance stress amplitude is $\Delta \sigma = 11\;{\text{MPa}}$, damage to the specimen occurs after 100 cycles of loading. When the disturbance stress amplitude is $\Delta \sigma = 15\;{\text{MPa}}$, damage to the specimen occurs after 70 cycles of loading. Obviously, there is a perturbation threshold for the disturbance stress amplitude at which damage to the structural plane rock occurs under the same static load stress.

**Figure 8:**
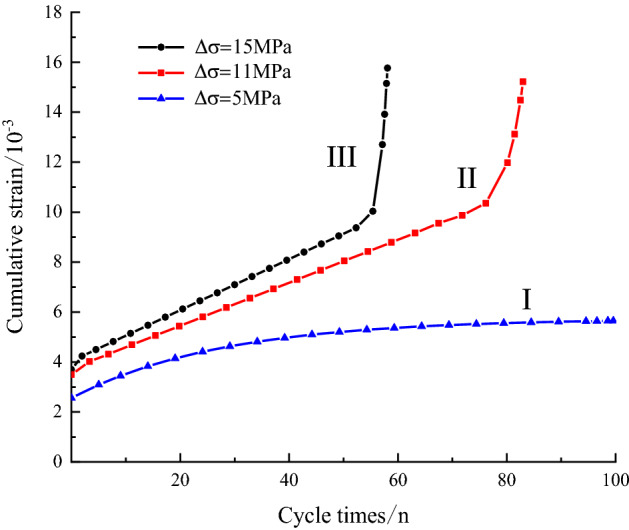
Corresponding relationship between cycle numbers and cumulative strain.

The axial effective plastic deflection strain corresponding to each plastic hysteresis loop at the upper limit stress in Fig. 7 is taken as the covariate of irreversible deformation, and a dotted line of the accumulated plastic strain with the number of perturbation cycles is plotted with the test data, as shown in Fig. 8. The plastic strain accumulation value and the number of perturbation cycles are roughly divided into two cases: one is that the rock material is not damaged when the perturbation stress amplitude is small, corresponding to the change trend shown in curve I, and the other is that the rock material is damaged when the perturbation stress amplitude is large, corresponding to the change trend shown in curve II and curve III. Therefore, to describe the two stress states of this curve, a perturbation threshold $\Delta \sigma_{\beta }$ is introduced. When the disturbance stress amplitude is less than the disturbance threshold, the plastic strain value tends to a stable value with the increase in cyclic loading and unloading cycles, and no damage occurs on the structural plane. Conversely, when the perturbed stress amplitude is greater than the perturbation threshold, the relationship curve between the plastic bias strain accumulation value and the number of cycles during the whole perturbed cyclic loading process has an S- shape. In the initial acceleration stage, the deformation rate is fast and a small part of the strain is produced. With an increasing number of cycles, the deformation rate becomes slow, and the amount of deformation is small. When the plastic strain accumulates to a certain extent due to the deformation rate increasing suddenly and the cumulative strain increasing rapidly, the failure and instability of the structural plane can be judged.

### Progressive damage mechanism of the structural plane under dynamic disturbance

**Figure 9:**
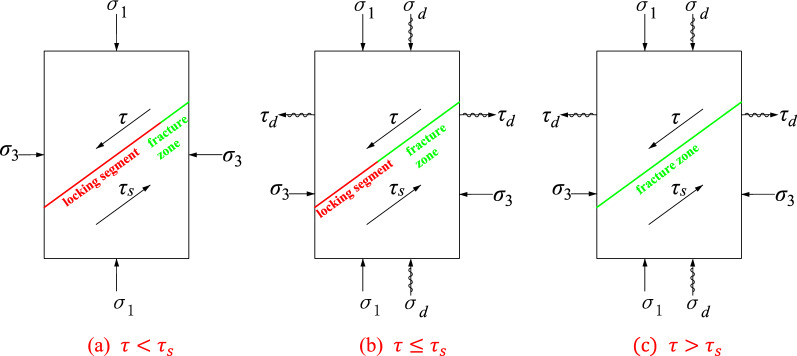
Schematic diagram of the structural plane damage mechanism.

For deep structural planes in the yield strength limit, shear damage along the structural plane is a common damage mode. Under the combined effect of multiple factors, such as the ground stress and self-weight of the rock body, the lithology on both sides of the structure surface will respond differently to the stress, resulting in localized displacement and cracking at the lithological interface. The cohesion in these rupture zones disappears and only friction exists, but the structural plane can still reach a self-stabilizing state, indicating that the shear strength provided by the locked section and the rupture zone is sufficient to balance the shear stress applied to the structural plane without damage. $\tau$ is the shear strength provided by the locked section and the rupture zone, and ${\tau }_{s}$ is the shear stress applied to the structural plane, as shown in Fig. 9a. When the amplitude of the periodic disturbance stress on the structural plane exceeds the disturbance threshold, the locking section part is further damaged, the length is continuously reduced, and the rupture length gradually increases. However, this transformation does not necessarily cause immediate structural plane instability but leads to overall damage, which is manifested by the accumulation of plastic strain on the structural plane and the reduction in shear stress, as shown in Fig. 9b. With the continuous loading of the perturbation, when the plastic strain accumulation on the structural plane is at some critical value, the impedance provided by the residual locking section cannot balance the shear stress inside the structural plane, and the structural plane of the residual locking section will rupture rapidly, which is macroscopically manifested as accelerated shear damage, as shown in Fig. 9c. When the amplitude of the periodic disturbance stress is less than the disturbance threshold, the disturbance stress makes the locking section undergo only elastic deformation, and the rupture zone is further damaged under the action of the disturbance tensile stress, resulting in the reduction of the friction force. The macroscopic performance of the plastic strain accumulation reaches stability to a certain degree, and the rock material does not undergo damage. From the microscopic point of view, when the structural plane rock body is subjected to periodic dynamic disturbance, the stress at the crack tip of the structural plane tensile damage is concentrated, and the grain structure of the structural plane rock body at the crack tip undergoes microdislocation and slip. This irreversible dislocation and slip is accumulated and superimposed under the action of multiple dynamic disturbances, resulting in the accumulation of plastic strain. When the disturbance stress amplitude is greater than the disturbance threshold, with the further expansion of the deformation, the dynamic stress intensity factor at the tip of the fine crack at the locking section increases to the critical value of crack expansion, and the crack will continuously open, expand, penetrate and connect to form macroscopic cracks and cause damage to the structural plane.

## Structural plane principal structure model considering perturbation threshold

The creep curve of rock under static force is mainly divided into three stages: transient creep, steady-state creep, and accelerated creep, and the cumulative plastic strain curve of fractured rock under perturbed cyclic loading is mainly divided into an initial acceleration stage, isometric stage, and abrupt acceleration stage. There are similarities between these two curves. To more accurately describe the three-stage deformation and damage process of structural plane rock under perturbed cyclic loading and unloading, this paper draws on the combined creep intrinsic model of rocks, equates the perturbed cyclic loading three-stage deformation pattern with the three-stage creep pattern, and regards the number of cycles as creep time to establish a nonlinear rheological intrinsic model of structural plane rock under perturbed cyclic dynamic loading.

### Establishment of nonlinear viscous acceleration elements

The Burgers model can effectively describe the first and second stages of the rock creep process, but because the Burgers model uses ideal linear elements, it is difficult to describe the accelerated creep phase of the rock, so a nonlinear accelerated element combination model is needed to describe its accelerated creep characteristics. There are two ways to build a nonlinear rheological model for rocks: (i) replacing the original linear components with new nonlinear components, and (ii) adopting new theories such as fracture and loss mechanics theory and internal time theory.

**Figure 10:**
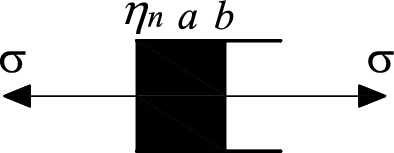
Schematic diagram of nonlinear viscous components.

According to the first approach, a new nonlinear viscous element is proposed in this paper, as shown in Fig. 10, whose instanton equation is

8 $$\sigma = \eta_{n} e^{a} \frac{{\mathop \varepsilon \limits^{ \bullet } }}{{bt^{b - 1} }}$$

where ${\eta }_{n}$ is the coefficient of viscosity; a is the coefficient of viscous elements; b is the rheological index, reflecting the rapidity of the accelerated creep rate of the rock; and t is the creep time.

Integrating Eq. (8), the creep equation for the nonlinear viscous accelerating element is obtained as

9 $$\varepsilon = \frac{\sigma }{{\eta_{n} e^{a} }}t^{b}$$

**Figure 11:**
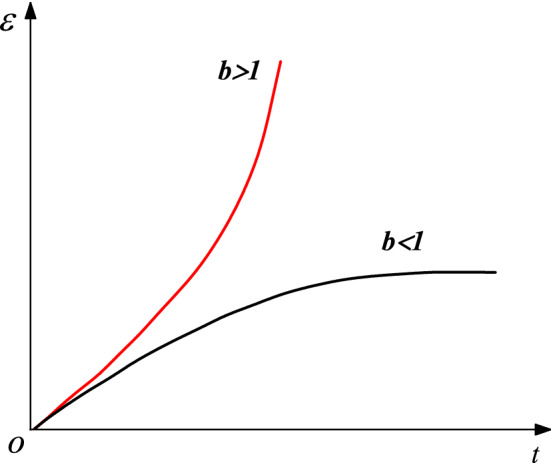
Nonlinear rheological curves for different values of parameter b.

The curves in Fig. 11 represent the rheological trends of the structural plane rock body in the acceleration phase when the rheological index b in Eq. (9) is taken as $b<1$ or $b>1$, respectively. The figure shows that only $b>1$ can reflect the nonlinear increase in both the strain and rate of the structural plane rock mass with time. Additionally, the above-mentioned element can be used to describe the nonlinear accelerated deformation of the structural plane rock mass.

**Figure 12:**
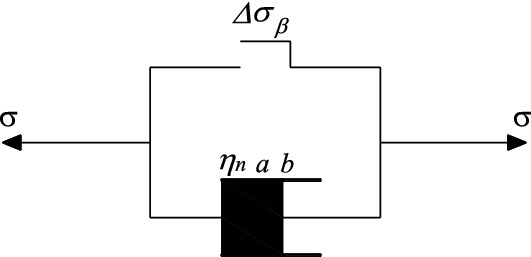
Nonlinear viscoplastic model.

Connecting it in parallel with a plastic element, as shown in Fig. 12

When the perturbed stress amplitude is less than the perturbation threshold, $\varepsilon =0$; when the perturbed stress amplitude is greater than the perturbation threshold, $\varepsilon =\frac{\sigma }{{\eta }_{n}{e}^{a}}{t}^{b}$.

### Modelling of structural plane strain

**Figure 13:**
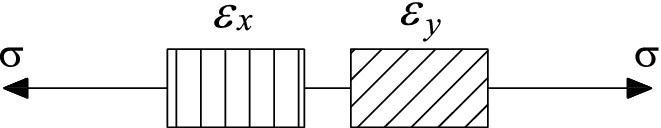
Model of structural plane strain.

When the structural plane is subjected to a compressive load, the deformation of the structural plane is divided into two parts: one part is the closed strain generated by the compression of the structural plane, and the other part is the shear strain generated along the structural plane. Therefore, two components are used to represent these two parts of strain, as shown in Fig. 13. The structural plane closure strain is calculated as:

10 $$\varepsilon_{x} = \varepsilon_{0} \left[ {1 - \exp \left( { - \frac{{\sigma \cos^{2} \beta }}{{\varepsilon_{0} E_{0} }}} \right)} \right]\cos \beta$$

where ${\varepsilon }_{0}=0.02$ is the maximum closure strain of the structural plane, ${E}_{0}=1980$ is the structural plane closure deformation parameter—the structural plane closure modulus (MPa), and $\sigma$ is the stress on the rock body of the structural plane.

According to the intrinsic relationship of the structural plane under shear load, the shear strain can be deduced as:

11 $$\varepsilon_{y} = \frac{{\sigma \sin^{2} \beta \cos \beta }}{{k_{s} L}}$$

${K}_{s}=2$ is the tangential stiffness of the structural plane (Pa/m), and L is the height of the rock specimen.

### Nonlinear 8-element creep instanton model

**Figure 14:**
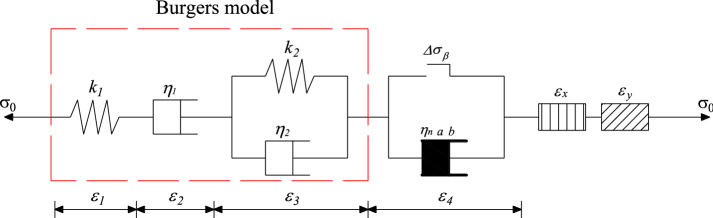
Nonlinear 8-element creep instanton model.

The above components and the Burgers model are connected in series, as shown in Fig. 14, to obtain a creep intrinsic model that can fully describe the 15° fractured rock mass under cyclic loading.

According to the series–parallel relationship between the components in the figure, the intrinsic relationships among all the parts can be obtained as

12 $$\left\{ \begin{gathered} \varepsilon = \varepsilon_{1} + \varepsilon_{2} + \varepsilon_{3} + \varepsilon_{4} + \varepsilon_{x} + \varepsilon_{y} \hfill \\ \sigma = \sigma_{1} = \sigma_{2} = \sigma_{3} = \sigma_{4} = \sigma_{x} = \sigma_{y} \hfill \\ \sigma_{1} = k_{1} \varepsilon_{1} \hfill \\ \sigma_{2} = \eta_{1} \dot{\varepsilon }_{2} \hfill \\ \sigma_{3} = k_{2} \varepsilon_{3} + \eta_{2} \dot{\varepsilon }_{3} \hfill \\ \sigma_{4} = \eta_{n} e^{a} \frac{{ \dot{\varepsilon }_{4} }}{{bt^{b - 1} }} \hfill \\ \varepsilon_{x} = \varepsilon_{0} \left[ {1 - \exp \left( { - \frac{{\sigma_{x} \cos^{2} \beta }}{{\varepsilon_{0} E_{0} }}} \right)} \right]\cos \beta \hfill \\ \varepsilon_{y} = \frac{{\sigma_{y} \sin^{2} \beta \cos \beta }}{{k_{s} L}} \hfill \\ \end{gathered} \right.$$

where ${\sigma }_{1}$, ${\sigma }_{2}$, ${\sigma }_{3}$, and ${\sigma }_{4}$ are the stresses of the elastomer, viscous body, viscoelastic body, and nonlinear viscoelastic body respectively. ${\varepsilon }_{1}$, ${\varepsilon }_{2}$, ${\varepsilon }_{3}$, and ${\varepsilon }_{4}$ are the strains of the elastomer, viscous body, viscoelastic body, and nonlinear viscoelastic body, respectively. ${k}_{1}$ and ${k}_{2}$ are the moduli of elasticity of the elastomer. ${\eta }_{1}$ and ${\eta }_{2}$ are the coefficients of viscosity of the viscous body. $\dot{{\varepsilon }_{2}}$, $\dot{{\varepsilon }_{3}}$, and $\dot{{\varepsilon }_{4}}$ are the first-order derivatives of ${\varepsilon }_{2}$, ${\varepsilon }_{3}$, and ${\varepsilon }_{4}$ respectively.

From the superposition principle, the creep equation of the intrinsic model of the jointed rock can be obtained as

13 $$\varepsilon = \left\{ \begin{gathered} \frac{{\sigma_{0} }}{{k_{1} }} + \frac{{\sigma_{0} }}{{\eta_{1} }}t + \frac{{\sigma_{0} }}{{k_{2} }}\left[ {1 - \exp \left( { - \frac{{k_{2} }}{{\eta_{2} }}t} \right)} \right] + \hfill \\ \varepsilon_{0} \left[ {1 - \exp \left( { - \frac{{\sigma_{0} \cos^{2} \beta }}{{\varepsilon_{0} E_{0} }}} \right)} \right]\cos \beta + \frac{{\sigma_{0} \sin^{2} \beta \cos \beta }}{{k_{s} L}} \quad \left( {\Delta \sigma \le \Delta \sigma_{\beta } } \right) \hfill \\ \frac{{\sigma_{0} }}{{k_{1} }} + \frac{{\sigma_{0} }}{{\eta_{1} }}t + \frac{{\sigma_{0} }}{{k_{2} }}\left[ {1 - \exp \left( { - \frac{{k_{2} }}{{\eta_{2} }}t} \right)} \right] + \hfill \\ \varepsilon_{0} \left[ {1 - \exp \left( { - \frac{{\sigma_{0} \cos^{2} \beta }}{{\varepsilon_{0} E_{0} }}} \right)} \right]\cos \beta + \frac{{\sigma_{0} \sin^{2} \beta \cos \beta }}{{k_{s} L}} + \frac{{\sigma_{0} }}{{\eta_{n} e^{a} }}t^{b} \quad \left( {\Delta \sigma > \Delta \sigma_{\beta } } \right) \hfill \\ \end{gathered} \right.$$

Because the creep time is equivalent to the number of cycles, the cumulative strain as a function of the number of cycles can be obtained by replacing *t* with *n* as

14 $$\varepsilon = \left\{ \begin{gathered} \frac{{\sigma_{0} }}{{k_{1} }} + \frac{{\sigma_{0} }}{{\eta_{1} }}n + \frac{{\sigma_{0} }}{{k_{2} }}\left[ {1 - \exp \left( { - \frac{{k_{2} }}{{\eta_{2} }}n} \right)} \right] + \hfill \\ \varepsilon_{0} \left[ {1 - \exp \left( { - \frac{{\sigma_{0} \cos^{2} \beta }}{{\varepsilon_{0} E_{0} }}} \right)} \right]\cos \beta + \frac{{\sigma_{0} \sin^{2} \beta \cos \beta }}{{k_{s} L}} \quad \left( {\Delta \sigma \le \Delta \sigma_{\beta } } \right) \hfill \\ \frac{{\sigma_{0} }}{{k_{1} }} + \frac{{\sigma_{0} }}{{\eta_{1} }}n + \frac{{\sigma_{0} }}{{k_{2} }}\left[ {1 - \exp \left( { - \frac{{k_{2} }}{{\eta_{2} }}n} \right)} \right] + \hfill \\ \varepsilon_{0} \left[ {1 - \exp \left( { - \frac{{\sigma_{0} \cos^{2} \beta }}{{\varepsilon_{0} E_{0} }}} \right)} \right]\cos \beta + \frac{{\sigma_{0} \sin^{2} \beta \cos \beta }}{{k_{s} L}} + \frac{{\sigma_{0} }}{{\eta_{n} e^{a} }}n^{b} \quad \left( {\Delta \sigma > \Delta \sigma_{\beta } } \right) \hfill \\ \end{gathered} \right.$$

## Model validation and parameter identification

**Figure 15:**
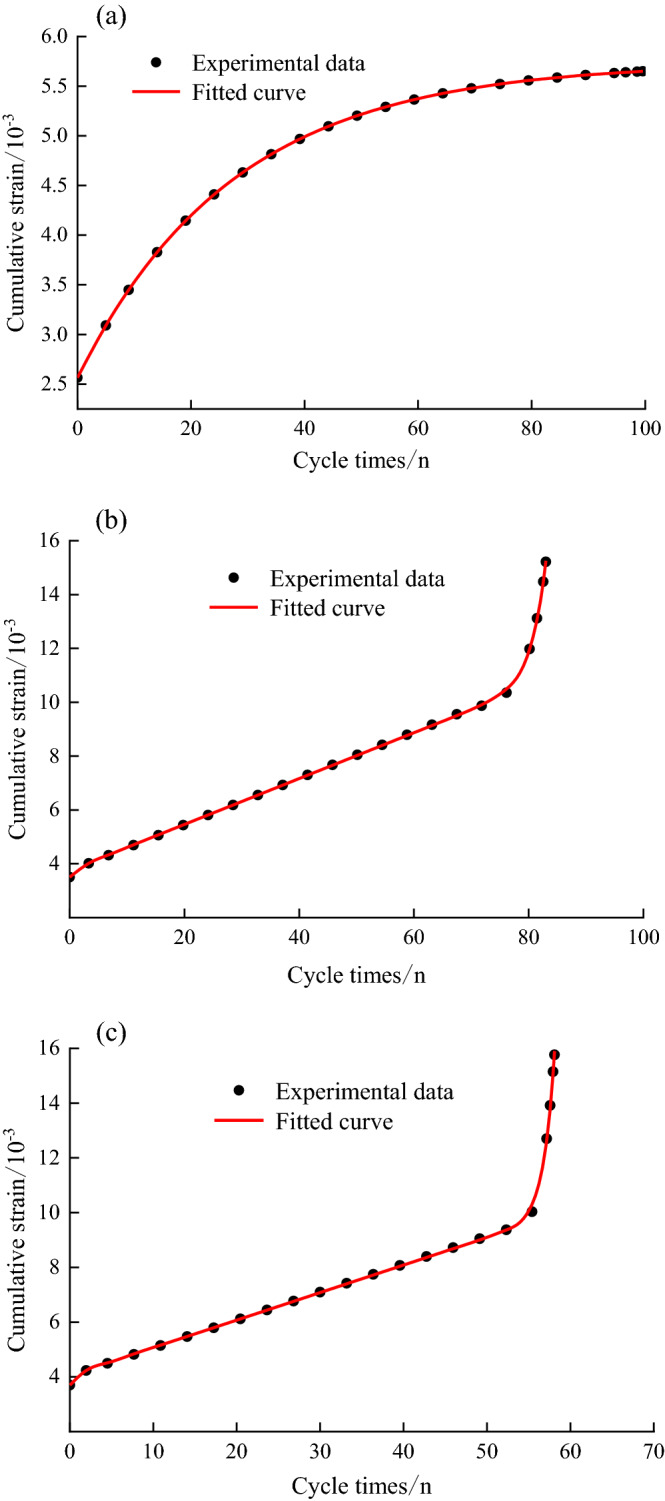
Fitting effect of the creep principal structure model: (**a**) $\sigma_{max} = 30\;{\text{MPa}}$ and $\Delta \sigma = 5\;{\text{MPa}}$, (**b**) $\sigma_{\max } = 36\;{\text{MPa}}$ and $\Delta \sigma = 11\;{\text{MPa}}$, (**c**) $\sigma_{\max } = 40\;{\text{MPa}}$ and $\Delta \sigma = 15\;{\text{MPa}}$.

Currently, the least squares method is one of the most popular methods used in the identification of model parameters for nonlinear problems, and one of the most commonly used is the Levenberg‒Marquardt (L–M) algorithm. The L–M algorithm is an improved nonlinear optimization algorithm between Newton's method and the gradient descent method, which not only provides the fast convergence of Newton's method and the global search property of the gradient descent method at the same time but also overcomes the disadvantage that the singular matrix cannot continue to be iterated, making it perform well in solving nonlinear problems. However, the L–M algorithm has a high requirement of the reasonableness of the initial value, and if the initial value given has a large deviation, it will lead to a large error in the recognition effect. Therefore, this paper adopts the 1stOpt software independently developed by 7D-Soft High Technology Inc for parameter identification. This software is a world leader in the fields of nonlinear regression and curve fitting; in particular, its built-in Universal Global Optimization algorithm solves the dependence of the L–M algorithm on the initial value; that is, the user does not need to determine the initial value, as it is randomly given by 1stOpt. Compared with other algorithms, the final fitting result of 1stOpt has higher precision. The model parameters and fitting results were obtained for each upper-stress level as shown in Table 2 and Fig. 15, by identifying the parameters of the accumulated deformation data during the whole process of cyclic dynamic loading of the 15° joint rock mass under different disturbance stress amplitudes. From the analysis of the results, it can be seen that the theoretical curves of the model and the experimental data are in good agreement and can reflect the whole process of the accumulated strain of the structural plane rock under cyclic loading, and the correlation coefficients ${R}^{2}$ are higher than 0.99, which indicates the correctness and rationality of the 8-element creep intrinsic model proposed in this paper.

**Table 2:** Results of fitting parameters for the creep principal structure model.

Upper limit stress /MPa	$k_{1}$/ GPa	$\eta_{1}$/(GPA·*h*)	$k_{2}$/GPa	$\eta_{2}$/(GPa·h)	$\eta_{n}$/(GPa·h)	*a*	*b*	*R*^*2*^
30	14.1674	20,090,351	11.3524	315.5032				0.9999
36	12.0892	493.34	163.3885	238.325	4.8763	147.0997	33.1388	0.9998
40	13.6236	497.34	136.3434	106.3419	1.2754	188.9325	46.0481	0.9995

## Conclusions

The strength effect of structural planes of a deep rock mass under blasting load is analysed, a stress analysis model of the structural plane under disturbance is established, and a guideline for the occurrence of shear damage of the structural plane under dynamic and static loading is given.

The periodic dynamic disturbance test shows that at the same ultimate static stress level, shear damage occurs on the structural plane under different values of disturbance stresses. When the perturbed stress amplitude is greater than the perturbation threshold, the accumulated plastic strain cannot be stabilized at a certain limit value but increases infinitely until damage, and the overall S-shaped trend is observed.

According to the instability of the structural plane of the rock mass, we propose the influence of the locking segment and fracture zone on the structural plane stability and reveal the damage mechanism in the process of the progressive damage of rock structural plane under periodic dynamic perturbation.

Considering the number of cycles as equivalent to creep time, an element that can simulate the nonlinear acceleration phase of the structural plane rock mass is developed, a nonlinear rheological instanton model of the structural plane rock mass under perturbed cyclic dynamic loading is established, and its creep instanton equation is derived.

The proposed intrinsic model is verified by disturbance test data of coarse-grained sandstone rock containing a structural plane, and the results show that the test data fit the theoretical curve of the model well, verifying the rationality and applicability of the proposed model.

## Supplementary Information

Supplementary Information.

## Data Availability

All data generated or analysed during this study are included in this published article [and its supplementary information files].
